# Leprosy

**Published:** 1885-07

**Authors:** 


### Leprosy.

Drs. G. H. Fox, of New York, and Graham, of Toronto (*Medical Record*), after a visit to Tracadie, in New Brunswick, where there are, at present, twenty-four inmates confined in the lazaretto, which is under the charge of the Sisters of Charity, submit the following propositions :

1. Leprosy is a constitutional disease, and in certain cases appears to be hereditary.
2. It is undoubtedly contagious by inoculation.
3. There is no reason for believing that it is transmitted in any other way.
4. Under certain conditions a person may have leprosy and run no risk of transmitting the disease.
5. It is not so liable to be transmitted to others as syphilis in its early stage. There is no relation between the two diseases.
6. Leprosy is usually a fatal disease, its average duration being from ten to fifteen years.
7. In rare instances there is a tendency to recover after the disease has existed many years.
8. There is no valid reason for pronouncing the disease incurable.
9. Judicious treatment improves the condition of the patient, and often causes a disappearance of the symptoms.
10. There is ground for the hope that an improved method of treatment will in time effect the cure of leprosy, or at least it will arrest and control the disease.

